# Fractal and multifractal analyses of bipartite networks

**DOI:** 10.1038/srep45588

**Published:** 2017-03-31

**Authors:** Jin-Long Liu, Jian Wang, Zu-Guo Yu, Xian-Hua Xie

**Affiliations:** 1 Key Laboratory of Intelligent Computing and Information Processing of Ministry of Education and Hunan Key Laboratory for Computation and Simulation in Science and Engineering, Xiangtan University, Xiangtan, Hunan 411105, China; 2 School of Mathematical Sciences, Queensland University of Technology, GPO Box 2434, Brisbane, Q4001, Australia

## Abstract

Bipartite networks have attracted considerable interest in various fields. Fractality and multifractality of unipartite (classical) networks have been studied in recent years, but there is no work to study these properties of bipartite networks. In this paper, we try to unfold the self-similarity structure of bipartite networks by performing the fractal and multifractal analyses for a variety of real-world bipartite network data sets and models. First, we find the fractality in some bipartite networks, including the *CiteULike, Netflix, MovieLens (ml-20m*), *Delicious* data sets and (*u, v*)-flower model. Meanwhile, we observe the shifted power-law or exponential behavior in other several networks. We then focus on the multifractal properties of bipartite networks. Our results indicate that the multifractality exists in those bipartite networks possessing fractality. To capture the inherent attribute of bipartite network with two types different nodes, we give the different weights for the nodes of different classes, and show the existence of multifractality in these node-weighted bipartite networks. In addition, for the data sets with ratings, we modify the two existing algorithms for fractal and multifractal analyses of edge-weighted unipartite networks to study the self-similarity of the corresponding edge-weighted bipartite networks. The results show that our modified algorithms are feasible and can effectively uncover the self-similarity structure of these edge-weighted bipartite networks and their corresponding node-weighted versions.

It is an indisputable fact that complex networks play a very critical role in characterizing complicated dynamics systems in nature and society. Empirical analyses have shown that many common characteristics and phenomena can be discovered from complex networks, e.g. small-world character^1^, scale-free property^2^, and self-similarity^3^. However, most of these pioneering works only focus on the unipartite networks, also called the classical networks or one-mode networks, which have only one class of nodes. Bipartite network, as a special kind of complex networks, has also attracted a great deal of attention from researchers in the fields of scientific research, engineering application, e-commerce, etc. The difference with the unipartite networks is the fact that the nodes of a bipartite network can be separated into two classes and its edges exist only between nodes of different classes. In real world, there are many systems, which can be modeled naturally by a bipartite network, such as the metabolic network^4^, the human sexual network^5^, actor-movie network^1^,^6^, scientist-paper network^6^,^7^, web-user network^8^, and so on. In addition, it is worth mentioning in particular that Guillaume *et al*.^9^,^10^ found that all complex networks have a nontrivial underlying bipartite structure.

Similar to the unipartite networks, many researchers studied the fundamental topological and statistical properties of bipartite networks, including the clustering coefficient^11^,^12^, average distance^12^, degree distribution^12^,^13^, modularity and community detection^14^,^15^, and evolving models^10^,^16^,^17^,^18^,^19^,^20^. In order to analyze the bipartite networks in a systematic way, Latapy *et al*. proposed an extension of the most basic notions used to analyze unipartite networks to the bipartite networks^12^. Although a lot of research works have been done on the study of bipartite networks, there is no a systematic framework for it compared with the unipartite networks. It is well known that, after the small-world character and scale-free property, self-similarity has become the third basic characteristic of complex networks. Based on the self-similarity of fractal geometry, Song *et al*.^3^ generalized the box-counting method and used it in the field of unipartite networks. They found that many complex networks such as the World-Wide-Web, social networks, protein-protein interaction networks (PINs), and cellular networks consist of self-repeating patterns. They characterized the self-similarity of these unipartite networks by the fractal dimensions calculated from the generalized box-counting method. They also noticed that not all unipartite networks show the clear self-similarity. More specifically, some unipartite networks show a shifted power-law (modified power-law or Mandelbrot’s law) behaviour or a pure exponential decay. So far, many algorithms have been proposed to calculate the fractal dimension of unipartite networks and then to study their self-similarity^21^,^22^,^23^,^24^,^25^,^26^,^27^. An improvement algorithm called the random sequential box-covering (RSBC) method, which is a modified version of the original method introduced by Song *et al*.^21^, was used to study the skeleton and fractal scaling property in scale-free networks^22^. Recently, our group adopted the RSBC algorithm to calculate the fractal dimensions of a family of fractal networks^28^. Our results showed that the fractal dimensions calculated by the algorithm coincide with the theoretical ones perfectly. Then we applied the RSBC algorithm to study the fractal property of the recurrence network constructed from fractional Brownian motions^29^. We found that the fractal dimension of the associated recurrence network obtained from the algorithm is very close to that of the graph of the fractional Brownian motions. In addition, Wei *et al*.^30^ proposed an improved box-covering algorithm for edge-weighted unipartite networks (BCANw). They showed that the BCANw is efficient to study the fractal property of edge-weighted unipartite networks.

The fractal analysis can help us to reveal the self-similarity of complex networks, but it is inadequate for some complex systems by a single fractal dimension. As a natural generalization of fractal analysis, the multifractal analysis may show more powerful than fractal analysis for some real-world fractal objects. The multifractal analysis has been widely applied in various fields such as financial modeling^31^,^32^, biological systems^33^,^34^, and geophysical data analyses^35^,^36^. In recent years, the multifractal analysis has also been successfully introduced to complex networks. Studies indicated that the tool of multifractal analysis have a better performance than the fractal analysis on characterizing the complexity of complex networks^28^,^29^,^37^,^38^. Meanwhile, some algorithms have been proposed to calculate the mass exponents *τ(q*) and the generalized fractal dimensions *D(q*) of complex networks. In order to improve the efficiency of the multifractal analysis algorithm, we employed the sandbox (SB) algorithm for multifractal analysis of complex networks^39^. Compared with the previous algorithms, our SB algorithm is the most effective and feasible algorithm to study the multifractality of complex networks. Then, our group modified the SB algorithm to explore the multifractal properties of edge-weighted networks (the SBw algorithm)^40^. It was found that the SBw is efficient for multifractal analysis of edge-weighted unipartite networks.

However, all the above mentioned fractal and multifractal analyses are just performed for unipartite networks. With the advent of web 2.0, there are many available bipartite networks. After that, the users no longer merely passively browse web sites, they also become active participants. More specifically, users can add the tags or ratings to these objects which they have browsed, bought or watched. In the past decade, the bipartite network has been introduced to the recommender system and shows better performance than the classical recommendation algorithms such as global ranking method and collaborative filtering^41^,^42^,^43^,^44^,^45^. In this work, we try to reveal the self-similarity of bipartite networks. This prompted us to study their fractality and multifractality. Here, we study the fractal and multifractal properties of some real-world bipartite network data sets and bipartite network models. In addition, motivated by the network-based resource-allocation dynamics in recommender system, we give different weights for two types of nodes (see Node-weighted bipartite networks subsection) to capture the essential nature of the bipartite networks with two different classes of nodes. Recently, our group study the dynamic-sensitive centrality of nodes in temporal networks^46^. For the data sets with ratings, however, we construct the corresponding edge-weighted bipartite networks and then try to probe their fractal and multifractal behaviors. Although there are two existing algorithms (BCANw and SBw) for fractal and multifractal analyses of edge-weighted networks, they are not suitable for all edge-weighted networks. We know that the key idea of the two existing algorithms is that the box size is obtained by accumulating the different edge-weights between two nodes linked directly, so that we can conveniently find an appropriate range to perform the least square linear fit and then calculate their fractal and generalized fractal dimensions. But there are so few different edge-weights in some bipartite networks. For example, the *Netflix* data set (see Empirical data sets subsection) contain only five different rating scales from 1 to 5 with a step of 1. This is to say, there are not more than five effective statistical dots in the log-log plot. In this case, it is difficult for us to find an appropriate range to calculate the fractal and generalized fractal dimensions of these bipartite networks. So, here we improve the RSBC and SBw algorithms (we call them IRSBCw and ISBw respectively) to adapt to such edge-weighted bipartite networks.

## Bipartite Network

### Basic notions

A bipartite network (or bipartite graph) *G* is often denoted by a triplet *G* = (*U, O, E*), where *U* and *O* are two disjoint sets of nodes, and *E* ⊆ *U* × *O* is the set of edges. In this paper, we model all the bipartite networks as the “user-object” bipartite networks. Naturally, we denote the user node (top node) set as 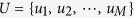 and the object node (bottom node) set as 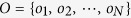. The edge *e*_*ij*_ ∈ *E* represents that the user *u*_*i*_ has already collected or rated the object *o*_*j*_. Therefore, a bipartite network can be fully described by a binary matrix *R*_*MN*_ = (*R*_*ij*_)_*MN*_. The *R*_*ij*_ = 1 if there exists an edge between the user *u*_*i*_ and the object *o*_*j*_, and the *R*_*ij*_ = 0 otherwise. In the rating data sets, however, the *R*_*ij*_ corresponds to the rating *r*_*ij*_ of the user *u*_*i*_ for the object *o*_*j*_. We call this network the edge-weighted bipartite network, where the edge-weight *w*_*ij*_ = *r*_*ij*_. The 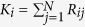 and 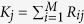 are the degrees of the user *u*_*i*_ and the object *o*_*j*_, respectively.

### Empirical data sets

In this paper, eight different real-world bipartite network data sets are used to study their fractality and multifractality. These bipartite network data sets have been widely used in various studies. The *CiteULike* (available at: http://www.citeulike.com) data set allows users to create their own collections of articles. The *Netflix* (available at: https://www.netflix.com/cn/) and *MovieLens (ml-20m)* (available at: http://files.grouplens.org/datasets/movielens/) data sets are two movie web sites allowing users to watch and rate movies. The higher the rating, the more user like it. The *Delicious* (available at: http://www.delicious.com/) data set is a bookmark web site allowing users collect and share bookmarks they interested in. The *Coactor* (available at: http://data.complexnetworks.fr/Bip/) data set is an actor-movie bipartite network, where each actor is linked to the movies he played in. The *Coauthor* (available at: http://data.complexnetworks.fr/Bip/) data set is an author-paper bipartite network, where each author is linked to the papers he/she published. The *Cooccurrence* (available at: http://data.complexnetworks.fr/Bip/) data set is a sentence-word bipartite network obtained from a version of the Bible, where each sentence is linked to the words it contains. The *Peer-to-Peer* (available at: http://data.complexnetworks.fr/Bip/) data set is a exchange bipartite network obtained by registering all the exchanges processed by a large server during 48 hours, where each peer is linked to the data. As mentioned above, all these empirical data sets are modelled as the “user-object” bipartite networks in this paper. Considering the limitation of the computational capacity of our computer, we only use a certain percentage of randomly selected records for some large-size data sets. The basic statistical properties of these data sets are listed in Table 1.

### Bipartite network models

#### Self-organized model

Collaboration networks is a particular class of social networks which widely exist in real world. Based on the preferential attachment concept put forward by Barabasi *et al*.^2^, Ramasco *et al*.^16^ proposed a growing and self-organizing bipartite network model for the collaboration networks. In this paper, we generate two bipartite networks according to the self-organized model. We obtain the first self-organized bipartite network (SOBNC) with parameters *t* = 4000, *n* = 4, and *m* = 2 when the two parameters *n* and *m* are constants. However, when the two parameters are random variables, we can generate the second self-organized bipartite network (SOBNV). In this case, the parameter *t* = 10000 and the other two parameters *n* and *m* are sampled randomly from two exponential distributions with averages 〈*n*〉 = 2.05 and 〈*m*〉 = 1.80, respectively.

#### Group-member model

However, there exists another class of bipartite networks which are composed of individual members and groups which gathered members with a common interest. Noh *et al*.^17^ proposed a growing bipartite network model to capture the growth rule of this class networks with a group structure. According to the different values of the selection probability *P*^*S*^ and the creation probability *P*^*C*^, they obtained the four possible different growth bipartite network models denoted by RV, RF, PV, and PF^17^. Here, we generate the four bipartite networks with parameters *m*_0_ = 3, *m* = 1, *ω* = 0.6, and the number of members *N*_0_ = 5000.

#### Nongrowing model

In the above two growing bipartite network models, the number of nodes and edges are increasing rapidly. However, based on a preferential rewiring process and a fitness distribution function, Ohkubo *et al*.^18^ proposed a nongrowing bipartite network model with the two sets of the fixed numbers of nodes and a fixed number of edges. Here we also assume that the degree distribution of the collaboration acts follows an exponential form in the initial network. Then, we consider the following two cases. First, we generate the initial network with the number of actors *M*_0_ = 10000, the number of collaboration acts *N*_0_ = 8000, the average degree of actors 〈*m*〉 = 3.0, and the average degree of collaboration acts 〈*n*〉 = 3.75. For the parameter *η*, we use the uniform fitness distribution given by *ρ(η*) = 1(0 ≤ *η* ≤ 1). In the second case, we use the initial network with *M*_0_ = 10000, *N*_0_ = 8000, 〈*m*〉 = 1.8, and 〈*n*〉 = 2.25. And the parameter *η* follows the exponential fitness distribution given by *ρ(η*) = *e*^−*η*^(0 ≤ *η* ≤ + ∞). The two cases are denoted by NonG-model1 and NonG-model2, respectively.

#### Growing bipartite model

In order to better understand the topological structure and dynamic law of bipartite networks, Guillaume *et al*.^10^ tried to capture the three main wanted properties (clustering, degree distribution, average distance) of real-world complex networks at the same time by their growing bipartite network model. In this paper, we generate bipartite networks with parameter *t* = 10000 and use three given degree distributions, including the exponential distribution with exponent *α* = 0.85 (G-model1), the power-law distribution with exponent *α* = 2.15 (G-model2), and the poisson distribution with exponent *α* = 2.8 (G-model3).

#### Mathematical model

Different with the above bipartite network models, Nacher^19^ *et al*. constructed a mathematical model to generate bipartite networks by considering the growth process and copy mechanism which generally exist in biological evolution. In this work, we generate such bipartite networks with parameters *l* = 4 and *t* = 9996. The other two parameters are set as: (1) *α*_*N*_ = 0.05 and *α*_*M*_ = 0.05 (M-model1); (2) *α*_*N*_ = 0.15 and *α*_*M*_ = 0.80 (M-model2); (3) *α*_*N*_ = 0.50 and *α*_*M*_ = 0.50 (M-model3); (4) *α*_*N*_ = 0.80 and *α*_*M*_ = 0.15 (M-model4).

#### Evolving model

Although there have been a variety of bipartite network models, Zhang *et al*.^20^ found that no one can describe the shifted power-law behavior of the degree distribution of online bipartite networks. Therefore, they proposed an evolving bipartite model to reveal the underlying mechanism of online bipartite networks. In this paper, we firstly generate the initial network with parameters *u*_0_ = 100, *v*_0_ = 100, and *e*_0_ = 10. Then we use this evolving model to generate three online bipartite networks. The other parameters are set as: (1) *t* = 10000, *m* = 1, *n* = 1, *b* = 1, and *c* = 1; (2) *t* = 10000, *m* = 2, *n* = 2, *b* = 2, and *c* = 5; (3) *t* = 10000, *m* = 1, *n* = 4, *b* = 5, and *c* = 3.

#### (*u, v*)-flower model

In 2007, Rozenfeld *et al*.^47^ proposed a (*u, v*)-flower model to reveal the growth mechanism of self-similarity of complex networks. Recently, we applied the SB algorithm to study the multifractal property of the (*u, v*)-flower network^39^. Here, we treat the (2,2)-flower network as a bipartite network. More specifically, when we consider the (2,2)-flower network of generation *n* + 1, the nodes of the *n*th generation (2,2)-flower network are modeled as the user nodes of the bipartite network and these new nodes naturally become the object nodes.

## Results and Discussion

### Fractal properties of bipartite networks

**Figure 1:**
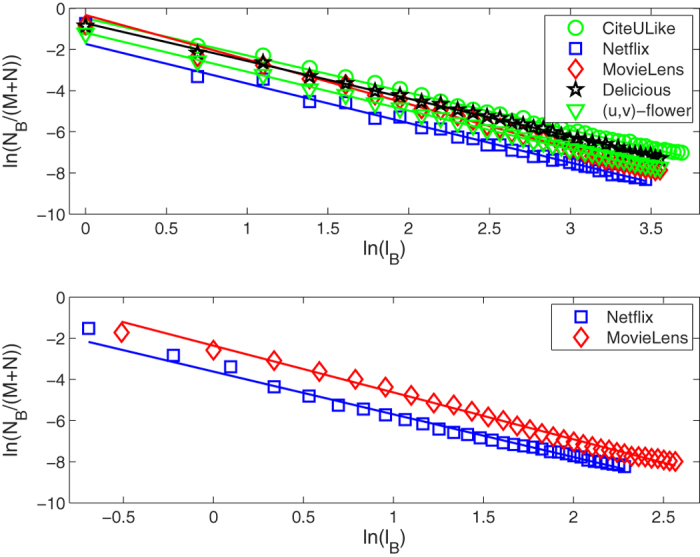
The log-log plot of *N*_*B*_(*l*_*B*_)/(*M* + *N*) versus *l*_*B*_ for the original bipartite networks (upper panel) and the two corresponding edge-weighted versions (lower panel). Solid line represents the linear fitting and the fractal dimension is the absolute value of the slope of the linear fit.

In this paper, we perform the fractal analysis for all these original bipartite networks by the RSBC method^22^. Here, we do not distinguish between the top nodes and the bottom nodes of bipartite networks. Figure 1 (upper panel) shows the apparent power-law relations in some bipartite networks between the number of boxes needed to cover the entire bipartite network and the box size, including the *CiteULike, Netflix, MovieLens (ml-20m), Delicious* data sets, and the 7th generation (*u, v*)-flower model with *u* = 2 and *v* = 2. For the *Netflix* and *MovieLens (ml-20m)* data sets with ratings, the higher the rating, the more user like it. So we construct two corresponding edge-weighted bipartite networks, where the edge-weight *w*_*ij*_ = *r*_*ij*_. And the value of *p* had better be a negative number in Eq. (4) (e.g. −1 given by Newman^7^). Here, we only consider *p* = −1 when calculating the distance of shortest path between nodes in the two edge-weighted bipartite networks. Then we apply the IRSBCw method (see Fractal analysis subsection) to study their self-similarity. From Fig. 1 (lower panel), we find that our IRSBCw method is feasible and can effectively unfold their self-similarity structure. This shows that the fractality exists in the two edge-weighted bipartite networks. The fractal dimension *d*_*B*_ is the absolute value of the slope of linear regression between *ln(N*_*B*_(*l*_*B*_)/(*M* + *N*)) and *ln(l*_*B*_), where *M* + *N* is the size of the bipartite network. The fractal dimensions of the six bipartite networks are 1.8106, 1.9212, 2.1617, 1.8310, 1.9056, 2.0769, and 2.2745, respectively. These results show that the fractal dimensions of original bipartite networks are slightly less than their corresponding edge-weighted versions.

**Figure 2:**
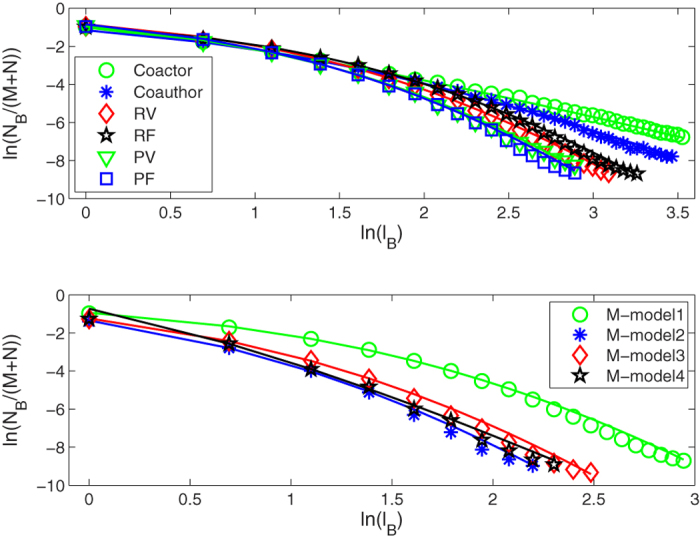
The log-log plot of *N*_*B*_(*l*_*B*_)/(*M* + *N*) versus *l*_*B*_ for different bipartite networks. Solid line represents the shifted power-law fit.

**Figure 3:**
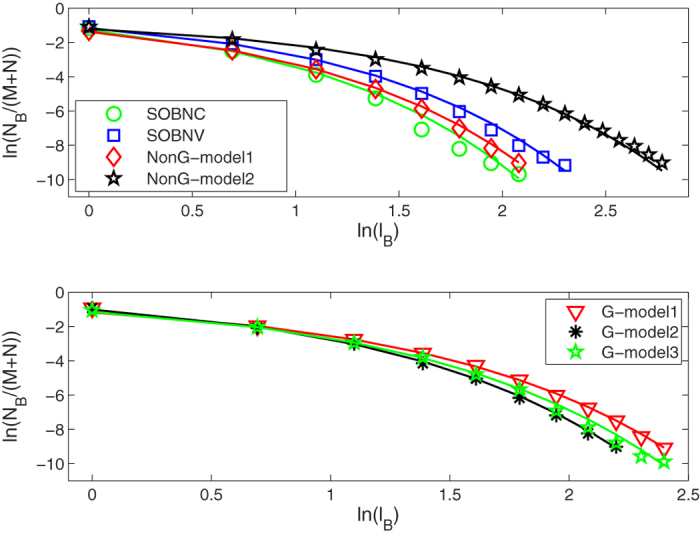
The log-log plot of *N*_*B*_(*l*_*B*_)/(*M* + *N*) versus *l*_*B*_ for different bipartite networks. Solid line represents the exponential fit.

As in Song *et al*.^3^, we here also find that some bipartite networks are lack of the clear self-similarity. From Fig. 2, we can observe the shifted power-law behavior in *Coactor, Coauthor*, Group-member model, and Mathematical model. We summarize these fitting results of shifted power-law in Table 2. As we can see from the Table 2, almost all of these bipartite networks have a relative large self-similarity exponent. This indicates that the decay of the number of boxes *N*_*B*_(*l*_*B*_) with the box size *l*_*B*_ is faster than a power-law. However, for the Self-organized model, Nongrowing model, and Growing model, our results show the pure exponential forms in Fig. 3. Their fitting results are *l*_*e*_ = 0.8097, 1.0803, 0.9177, 1.8136, 1.2698, 0.9908, and 1.1187, respectively. In other words, the values *l*_*e*_ of all these bipartite networks except for NonG-model2 approximately satisfy *l*_*e*_ ≈ 1. All these interesting phenomena are observed in Song *et al*.^3^. In addition, the diameters of the *Cooccurrence, Peer-to-Peer*, and Evolving model are too small so that we can’t perform fractal analysis for these bipartite networks.

Although our algorithms are proposed for bipartite networks, they can also be applied to calculate fractal dimensions of regular networks when bipartite networks are treated as unipartite networks made of two kind of nodes. For examples, we calculated the fractal dimensions of the metabolic network of *E. coli*^3^,^25^ and the 192 brain network clusters^26^, we obtained almost the same results as in previous works^3^,^25^,^26^ (the details are not shown here).

### Multifractal properties of bipartite networks

**Figure 4:**
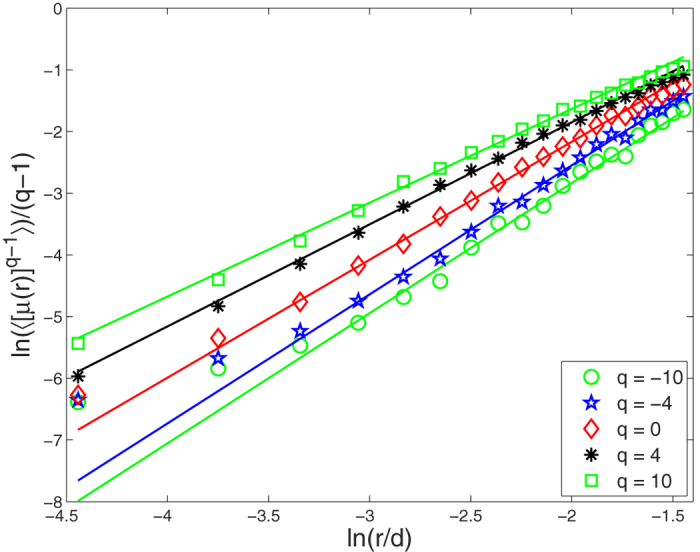
Linear regressions for calculating the generalized fractal dimensions of the *CiteULike* data set.

**Figure 5:**
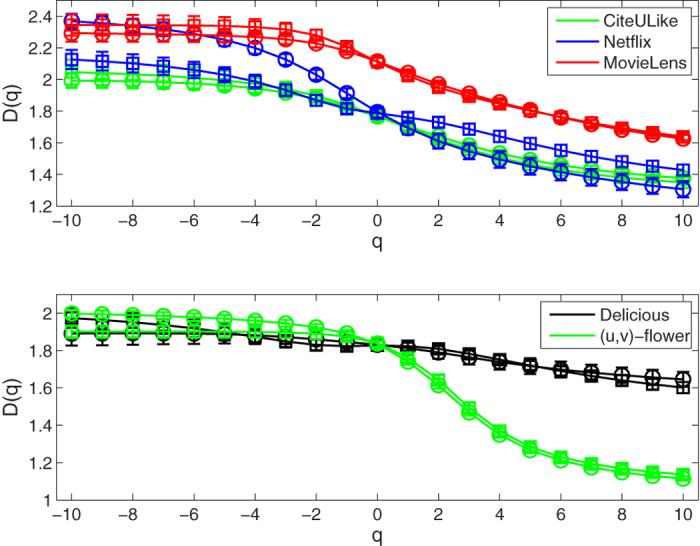
*D(q*) curves of bipartite networks. Circles and squares indicate the original bipartite networks and their corresponding node-weighted bipartite networks, respectively. Each error bar takes twice length to the standard deviation for all the results.

For these bipartite networks possessing the fractality, we further study their multifractality. Here, we set the range of the *q* values from −10 to 10 with a step of 1. We use the SB algorithm^39^ to perform multifractal analysis for these original bipartite networks. As an example, we show the linear regressions of the ln(〈[*μ(r*)]^*q*−1^〉)/(*q* − 1) vs ln(*r/d*) for the *CiteULike* data set in Fig. 4. As we can see from the Fig. 4, there are obvious power-law relation in the *CiteULike* data set for different *q* values. We then obtain the generalized fractal dimensions *D(q*) and their standard deviations by fitting linearly these power-law relation in the log-log plot. From Fig. 5, we find that the *D(q*) curves of these bipartite networks, including *CiteULike, Netflix, MovieLens (ml-20m), Delicious* data sets, and the 7th generation (*u, v*)-flower model with *u* = 2 and *v* = 2, are not straight lines. So the multifractality exists in these bipartite networks. In Fig. 5, each error bar takes twice length to the standard deviation. Meanwhile, we explore the multifractality of the corresponding node-weighted (see Node-weighted bipartite networks subsection) versions of these bipartite networks. The results of Fig. 5 show the existence of the multifractality in these node-weighted bipartite networks. From Fig. 5, we also find that the fractal dimensions *D*(0) of these original bipartite networks almost equal the fractal dimensions *D*(0) of their corresponding node-weighted versions. This is because that the fractal dimension is only related to the number of box needed to cover the entire network, not to the measure of each box.

**Figure 6:**
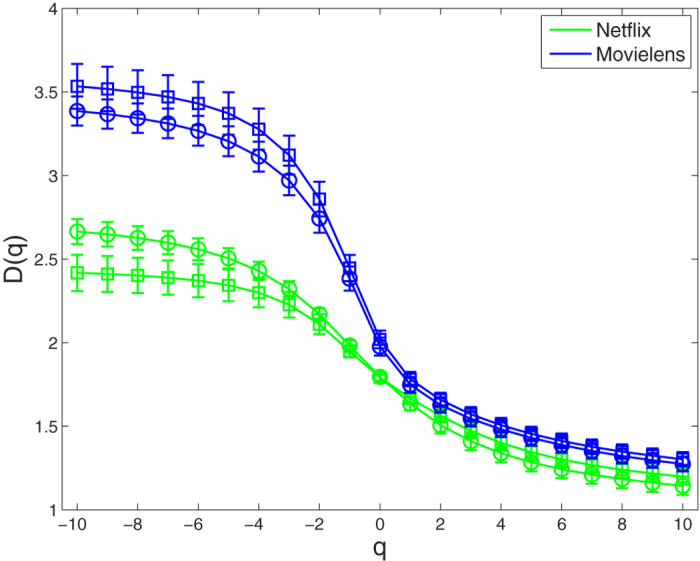
*D(q*) curves of bipartite networks. Circles and squares indicate the edge-weighted bipartite networks and their corresponding node-weighted bipartite networks, respectively. Each error bar takes twice length to the standard deviation for all the results.

For the *Netflix* and *MovieLens (ml-20m*) data sets with ratings, we apply the ISBw algorithm (see Multifractal analysis subsection) to detect the multifractal behavior of the edge-weighted bipartite networks and their corresponding node-weighted versions. We find that our ISBw algorithm is also feasible and can effectively reveal their multifractal property. As shown in Fig. 6, the results indicate that the multifractality exists in the two edge-weighted bipartite networks and their corresponding node-weighted versions. Here, we also observe that the fractal dimensions *D*(0) of the edge-weighted bipartite networks coincide with the fractal dimensions *D*(0) of their corresponding node-weighted versions.

## Conclusions

Compared with existing researches, our present work extended the research scope of bipartite networks. In this paper, we studied the self-similarity of bipartite networks. We applied the RSBC method to reveal the fractality of some real-world bipartite networks data sets and some bipartite network models. Our results showed that the self-similarity exists in some bipartite networks, such as *CiteULike, Netflix, MovieLens (ml-20m), Delicious* data sets, and the (*u, v*)-flower network model. For the *Netflix* and *MovieLens (ml-20m)* data sets with ratings, we construct two corresponding edge-weighted bipartite networks. We proposed the IRSBCw method to explore their self-similarity. We found that our IRSBCw method is feasible and can effectively unfold the self-similarity structure of these edge-weighted bipartite networks. The results indicated that the two edge-weighted bipartite networks possess the fractality. Meanwhile, we also noticed that not all bipartite networks show the obvious self-similarity. More specifically, the shifted power-law was observed in *Coactor, Coauthor*, Group-member model, and Mathematical model. However, the Self-organized model, Nongrowing model, and Growing model indicate the pure exponential forms. In addition, the diameters of the other bipartite networks are too small so that we can’t perform the fractal analysis for these bipartite networks.

Then we used the SB algorithm which is used to explore the multifractal properties of unipartite networks to detect the multifractal behavior of these original bipartite networks possessing the fractality. Our results of multifractal analysis show that the multifractality exists in these bipartite networks. In order to capture the essential nature of the bipartite networks with two different classes of nodes, we construct the node-weighted bipartite networks. These results indicated the existence of multifractality in these bipartite networks. At the same time, for the edge-weighted bipartite networks, we applied our ISBw algorithm to explore their multifractal property. We found that the ISBw algorithm is also feasible and can effectively reveal their multifractal properties. We observed the multifractal behavior in the two edge-weighted bipartite networks and their corresponding node-weighted versions. In addition, our IRSBCw and ISBw algorithms are also effective for fractal and multifractal analyses for unweighted unipartite networks.

## Methods

### Fractal analysis

Mandelbrot^48^ introduced the fractal idea in 1967. In fractal geometry, a fractal object is self-similar because it contains local parts similar to the whole^49^,^50^. In order to characterize complex fractal sets, many algorithms have been developed to calculate their fractal dimensions^48^,^49^,^50^. Based on the self-similarity of fractal geometry, the box-counting algorithm, which is often used to calculate the fractal dimension of fractal objects, was generalized by Song *et al*.^3^ and successfully applied to calculate the fractal dimension of complex networks and then to uncover the self-similar structure of complex networks. In the algorithm, for each value of the box size *l*_*B*_, we can approximately obtain the minimum number of boxes *N*_*B*_(*l*_*B*_) needed to tile the entire network. The fractal dimension *d*_*B*_ is then defined as


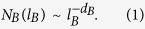


In practice, the fractal dimension *d*_*B*_ can be estimated by fitting the linear relationship between *N*_*B*_(*l*_*B*_) and *l*_*B*_ in a log-log plot. In addition, they also noticed an interesting phenomenon that not all complex networks show the obvious self-similarity in their Supplementary Information^3^. They found that the result of *N*_*B*_(*l*_*B*_) of the Internet network can be well fitted with a shifted power-law


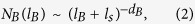


with *l*_*s*_ = 14.9 representing a cut-off and *d*_*B*_ = 8.5^3^. For *H. pylori* and *D. melanogaster* PINs, the fitting results are pure exponential forms


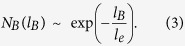


For edge-weighted unipartite networks, although Wei *et al*.^30^ has proposed the BCANw algorithm for fractal analysis of edge-weighted unipartite networks, the algorithm is not effective for all edge-weighted networks. In the BCANw algorithm, the key point is that the box size *l*_*B*_ is obtained by accumulating the different edge-weights between two nodes linked directly until it is more than the diameter of the network, so that we can obtain enough numbers of boxes to conveniently find an appropriate range for performing the least square linear fit and then calculating the fractal dimensions of edge-weighted unipartite networks. But there are so few different edge-weights in some bipartite networks. For example, the *Netflix* data set contain only five different kinds of rating scales from 1 to 5 with a step of 1. Consequently, there are only five different edge-weights in the corresponding edge-weighted bipartite network. In other words, there are not more than five valid statistical points in the log-log plot between the box size *l*_*B*_ and the minimum number of boxes *N*_*B*_(*l*_*B*_) needed to cover the entire network. In this case, it is hard for us to find an appropriate range to calculate the fractal dimensions of such bipartite networks. In addition, the diameters of these bipartite networks are relatively large. This leads to another imperfection that the sum of all different edge-weights of these bipartite networks is much less than their diameter. Based on the above considerations, the IRSBCw algorithm is proposed to adapt to such bipartite networks.

In the BCANw algorithm, the distance of shortest path between node *i* and node *j* is defined as ref. 30


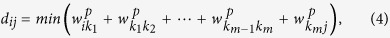


where *w*_*ij*_ is the edge-weight and *p* is a real number. The *p* = 0 means that the edge-weighted network reduces to the unweighted network. In real-world edge-weighted networks, there are two opposite meanings for other *p* values. On the one hand, the *p* > 0 represents that the larger edge-weight is, the further distance is. The real city network is such an example, where the edge-weight is the Euclidean distance between two cities. On the other hand, the *p* < 0 shows that larger edge-weight is, the less distance is. In the scientific collaboration networks, for example, the edge-weight corresponds to the number of papers coauthored by two scientists. Especially, Newman^7^ use the *p* = −1 to calculate the minimum distances between two scientists on a edge-weighted network. Different with the BCANw algorithm, we obtain box size *l*_*B*_ from the minimum edge-weight 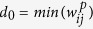 to the diameter *d* of the network with equal linearly step. The IRSBCw method for fractal analysis of edge-weighted bipartite networks can be described as follows:

**step 1** For a given *p*, obtain the minimum edge-weight *d*_0_ and the diameter *d* of the network by the Eq. (4).

**step 2** Obtain the box size *l*_*B*_ from *d*_0_ to *d* with equal linearly step, the step can be calculated according to the *d*_0_ and *d*.

**step 3** For different values of *l*_*B*_, the minimum number of boxes *N*_*B*_(*l*_*B*_) needed to tile the entire network can be approximately obtained by the classical RSBC method^22^.

From the above description we can see that the IRSBCw algorithm can also be applied in the unweighted bipartite networks, weighted and unweighted unipartite networks.

### Multifractal analysis

In the 1980’s, Grassberger and Halsey *et al*. introduced the multifractal analysis to systematically characterize the spatial heterogeneity of both theoretical and experimental fractal objects^51^,^52^. The fixed-size box-covering algorithm^52^ is one of the most common and important methods of multifractal analysis. For a given measures *μ* with support set 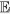 in a metric space, we consider the following partition sum


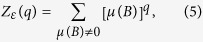


where 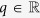 and the sum runs over all different nonempty boxes *B* of a given size *ε* in a box covering method of the support set 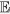. From the definition above, we can easily obtain *Z*_*ε*_(*q*) ≥ 0 and *Z*_*ε*_(0) = 1. The mass exponents *τ(q*) of the measure *μ* can be defined as


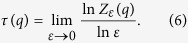


The generalized fractal dimensions *D(q*) of the measure *μ* are defined as


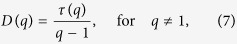


and


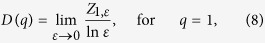


where *Z*_1,*ε*_ = ∑_*μ(B*)≠0_*μ(B*)ln*μ(B*). The linear regression of [ln*Z*_*ε*_(*q*)]/(*q* − 1) against ln*ε* for *q* ≠ 1 gives a numerical estimation of the generalized fractal dimensions *D(q*), and similarly a linear regression of *Z*_1,*ε*_ against ln*ε* for *q* = 1. In particular, the value *D*(0) is the box-counting dimension (or fractal dimension), *D*(1) is the information dimension, and *D*(2) is the correlation dimension. In addition, we can determine the multifractality of complex network by the shape of *D(q*) or *τ(q*) curve. More specifically, if the *τ(q*) or *D(q*) curve versus *q* is a straight line, the object is monofractal. However, if this curve is convex, the object is multifractal.

In order to easily obtain the generalized fractal dimensions *D(q*), Tél *et at.*^53^ introduced a sandbox algorithm. The main strength of the algorithm is that we can randomly choose a point on the fractal object as the center of a sandbox and then count the number of points in the sandbox. Results showed that the sandbox algorithm can give a better estimation of the *D(q*). The generalized fractal dimensions *D(q*) are defined as


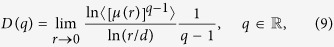


where 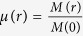 is the measure of the sandboxes with radius *r, M(r*) is the number of points in a sandbox with a radius of *r* and *M*(0) is the total number of points in the fractal object. The brackets 〈·〉 mean to take statistical average over randomly chosen centers of the sandboxes. The above equation also can be rewritten as


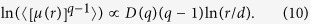


In practice, we often estimate numerically the generalized fractal dimensions *D(q*) by performing a linear regression of ln(〈[*μ(r*)]^*q*−1^〉)/(*q* − 1) against ln(*r/d*).

For unipartite networks, the measure *μ(r*) of each box is usually defined as the ratio of the number of nodes covered by the box and the total number of nodes in the entire network. In 2015, we applied the SB algorithm to study the multifractality of unipartite networks and found that this is the most effective and feasible algorithm^39^. However, there are two types of nodes in bipartite networks. To reflect the essential attribute of bipartite network with two types of nodes, we give node-weight (see Node-weighted bipartite networks subsection) to each node of bipartite network. At this point, the measure *μ(r*) of each box is the sum of the weights of nodes in this box.

As mentioned above, for the edge-weighted bipartite networks, we can obtain the radius *r* of boxes from the minimum edge-weight 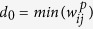 to the diameter *d* of the network with equal linearly step to improve the SBw algorithm proposed by Song *et al*.^40^. The steps of the algorithm are given as follows:

**step 1** For a given *p*, obtain the minimum edge-weight *d*_0_ and the diameter *d* of the network by the Eq. (4).

**step 2** Obtain the radius *r* of the sandbox from *d*_0_ to *d* with equal linearly step, the step can be calculated according to the *d*_0_ and *d*.

**step 3** For different values of *r*, the statistical average 〈[*μ(r*)]^*q*−1^〉 can be obtained by the classical SB algorithm^39^.

### Node-weighted bipartite networks

**Figure 7:**
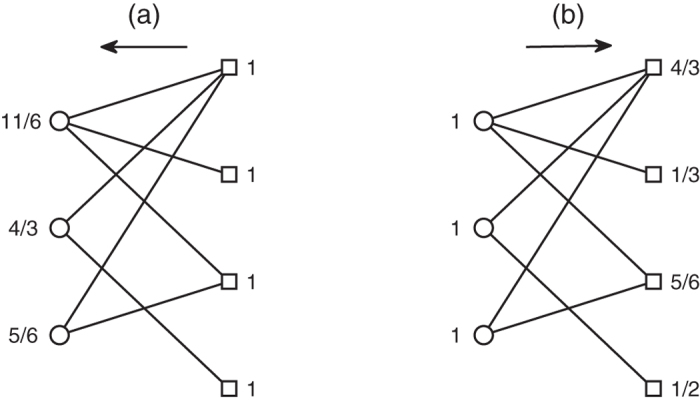
An example for calculating the node weight of bipartite networks. Circles and squares indicate the user and object nodes, respectively.

In recommender system, the amount of resource per node of bipartite network has a special significance^41^,^42^,^43^. The amount of resource per node means its recommending capacity. Initially, the initial resources of these objects which have already been collected by a target user are unit, otherwise they are zeros. We can then obtain the final resource of each object node after two steps of resource-allocation. Finally, only these object nodes with highest value of final resource may be recommended to the target user. During the process of resource-allocation, the amount of resource per node is not only related to its own degree, but also to the degree of its neighbor node. In other words, the more the number of users collecting a object, the more important the object is. In addition, if two users commonly collect a popular object (object node with large degree), the object do not distinguish the personal interest between the two users. This is to say, the objects with small degree can reflect user’s personalization more effectively. Similarly, only the users with small degree can capture the special attributes of object. So the amount of resource of a object (user) obtained from different users (objects) is different. Obviously, the object (user) should get more resources from the user (object) nodes with small degree. The difference with recommender system is that the initial resource of all nodes are unit in this paper. That is because we don’t need to get the finial resources for object nodes from the two steps of resource-allocation, we just need to know the relative recommending capacity of each object and user nodes of bipartite network. We take the relative recommending capacity of each node as its weight. After the normalization, the weight of the user node *u*_*i*_ is given by


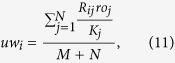


where *ro*_*j*_ is the initial resource of the object node *o*_*j*_. Also, the weight of the object node *o*_*j*_ is defined as


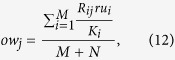


where *ru*_*i*_ is the initial resource of the user node *u*_*i*_. In Fig. 7, we give an example for calculating the node weight of each node of the bipartite network with *M* = 3 and *N* = 4. The weight of the user and object nodes can be obtained from the Fig. 7(a,b), respectively. For example, the weight of the first user node 
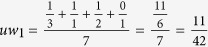
.

## Additional Information

**How to cite this article**: Liu, J.-L. *et al*. Fractal and multifractal analyses of bipartite networks. *Sci. Rep.* **7**, 45588; doi: 10.1038/srep45588 (2017).

**Publisher's note:** Springer Nature remains neutral with regard to jurisdictional claims in published maps and institutional affiliations.

## Figures and Tables

**Table 1:** Basic statistical properties of eight real-world bipartite network data sets.

Data set	User	Object	Num. of users	Num. of objects
*CiteULike*	User	Article	4465	4044
*Netflix*	User	Movie	11546	1772
*MovieLens*	User	Movie	10499	3665
*Delicious*	User	Bookmark	5064	11461
*Coactor*	Actor	Movie	12667	12881
*Coauthor*	Author	Paper	16400	19885
*Cooccurrence*	Sentence	word	13587	9264
*Peer-to-Peer*	Peer	Data	15906	17923

**Table 2:** The fitting results of shifted power-law for different bipartite networks.

	Coactor	Coauthor	RV	RF	PV	PF	M-model1	M-model2	M-model3	M-model4
*l*_*s*_	1.4354	3.6051	8.3736	10.0930	8.5403	8.0066	8.7968	4.0315	5.3113	1.1819
*d*_*B*_	2.1604	3.2223	6.6056	6.6107	7.2252	7.2572	7.3961	7.9852	8.0975	4.8727
